# Editorial: Asthma in Children and Adults – What Are the Differences and What Can They Tell Us About Asthma?

**DOI:** 10.3389/fped.2020.00141

**Published:** 2020-04-01

**Authors:** Steve Turner, John W. Upham

**Affiliations:** ^1^Child Health, University of Aberdeen, Aberdeen, United Kingdom; ^2^Faculty of Medicine, The University of Queensland, Princess Alexandra Hospital, Brisbane, QLD, Australia

**Keywords:** asthma, child, adult, difference, similarity

In the 1970s, asthma was an enigmatic condition with no agreed definition or diagnostic test, but which was nonetheless diagnosed and treated every day. Fast forward 50 years and the situation is mostly unchanged. Uncertainty about what asthma is has hampered development of new treatments. The care for other non-communicable diseases such as diabetes, leukemia, epilepsy, and inflammatory bowel disease has leapt ahead, whilst most patients with asthma are receiving the same treatment their parents were prescribed a generation ago, i.e., short acting beta agonists and inhaled corticosteroids. New treatments options have emerged for severe asthma treatment, but these impact on a relatively small proportion of people with asthma.

The subjective nature of asthma diagnosis causes both under- and over- diagnosis. This uncertainty results in preventable morbidity firstly due to treatment side effects where “asthma” is not present and secondly where effective treatment is not given.

It is now widely accepted that what is currently recognized as “asthma” is a syndrome (1) which describes a range of different pathologies which share common symptoms (i.e., wheeze, shortness of breath and cough) and common physiology (i.e., reversible airway obstruction). Over the years attempts to prise apart these different facets of asthma by stratifying have proven unhelpful; examples of well-meaning but ultimately unhelpful categorizations include atopic vs. non-atopic asthma, intrinsic vs. extrinsic asthma and intermittent vs. persistent asthma. Well-crafted efforts to describe asthma (or wheeze) phenotypes have proved convenient but ultimately flawed. For example, terms such as viral wheeze, multi trigger wheeze and the Asthma COPD Overlap Syndrome have been used to try and capture the potential overlap between “asthma” and other wheezing syndromes which are present early and later on in the life course. In children, viral wheeze and multi trigger wheeze are unstable phenotypes (2) (i.e., over time individuals viral wheeze develop multitrigger wheeze and vice versa) and in adults the “Dutch hypothesis” (3) remains hotly debated. The observation that viral wheeze (aka wheezy bronchitis) and asthma are both risk factors for COPD (4) further dampens enthusiasm for these phenotypes.

One phenotype which seems durable and appears in most attempts at cracking the asthma puzzle is childhood onset asthma (5–7). From a genetic perspective there are different variants associated with childhood onset asthma compared to other phenotypes (8) or adult onset asthma (9). “Childhood onset” is generally considered to include up to age 12 years but the persistently low lung function trajectory associated with asthma is present by 6 years of age (10, 11), and this suggests that “childhood onset” might be refined to onset by 5 years of age. Further recognition that asthma may be different between adult and children is seen in asthma guidelines which have different approaches to diagnosis and management of asthma in children and adults. When adult chest physicians and pediatric pulmonologists meet at international conferences, local educational events and transition clinics, there are often differences in approaches for adults and children. For example, adult physicians believe that they can improve asthma symptoms with treatment for rhinitis and gastro esophageal reflux, but this is not clearly seen in children. Aspirin-sensitive asthma is almost exclusively seen in adults. Children inhale lots of environmental exposures at school but do not demonstrate an equivalent of occupational asthma. Steroid-resistant asthma is much more commonly seen in adult than in children and severe asthma in preschool children is very uncommon (and likely to be a manifestation of something other than asthma). Adult-onset, eosinophilic asthma with nasal polyps in the absence of atopy is a well-recognized entity in adults, but is rarely seen in children.

Has the elusive first key to unlocking the asthma conundrum been staring us in the face all along? Could childhood-onset asthma vs. adult onset asthma(s) be the obvious first way to start dissecting asthma?

We challenged experts in asthma from around the world to compare and contrast asthma in adults and children. The author's challenge was not to simply reproduce the many guidelines for asthma diagnosis and management. Each article compared and contrasted asthma in adults and children within the following 11 domains: epidemiology (causation) (Dharmage et al.); epidemiology (life-course) (Trivedi and Denton); genetics (including gene environment interactions) (Morales and Duffy); diagnosis (Saglani and Menzie-Gow); monitoring (Gallucci et al.); treatment (chronic symptoms) (Chung and Paton); treatment (acute symptoms) (Chavasse and Scott); dysfunctional breathing (Connett and Thomas); severe asthma (Fleming and Heaney); primary care (Kaplan et al.); and transition (Withers and Green). Authors of each article included an adult chest physician and a pediatric pulmonologist (with the exception of primary care). After submitting their article, authors were asked to give a “score of similarity” from the perspective of their article using the following scale:

0 Asthma is a totally different condition in children and adults
1 There is ~10% overlap between childhood asthma and adult asthma
2 There is ~20% overlap between childhood asthma and adult asthma
3 There is ~30% overlap between childhood asthma and adult asthma
4 There is ~40% overlap between childhood asthma and adult asthma
5 There is ~50% overlap between childhood asthma and adult asthma
6 There is ~60% overlap between childhood asthma and adult asthma
7 There is ~70% overlap between childhood asthma and adult asthma
8 There is ~80% overlap between childhood asthma and adult asthma
9 There is ~90% overlap between childhood asthma and adult asthma
10 Asthma is the same condition in children and adults.

The resulting scores ranged between 4 and 8, Table 1. The domains of epidemiology (life-course), genetics and severe asthma were rated as having only 40–50% overlap between childhood and adult asthma. In contrast, all other domains which were scored were considered to have 70–80% overlap.

**Table 1:** This describes the 11 domains of asthma in which each article associated with this article compared and contrasted asthma in adults and children.

**Domain**	**Nationality of authors**	**Score of similarity**
1. Epidemiology (causation)	UK Australia	7
2. Epidemiology (life course)	US, Australia	4
3. Genetics (including gene environment interactions)	Spain, Australia	4
4. Diagnosis	UK	7
5. Monitoring	Italy	^*^
6. Treatment (chronic)	Australia UK	7
7. Treatment (acute)	UK	8
8. Dysfunctional breathing	UK	7
9. Severe asthma	UK	5
10. A primary care perspective	Canada, UK, Singapore	7
11. Transition from pediatric to adult services	Australia UK	^†^

*The table also states the nationality of authors and the authors “score of similarity” which rates the overlap between asthma in adults and children from the perspective of their article (higher score indicating greater overlap, see text for definition of score)*.

* *A score was not available. A score was not sought since the age range at transition is so narrow*.

So do these different perspectives give us any insight into how we can start to solve the asthma enigma? Well yes and no. The international experts who considered adult and childhood asthma from these different perspectives are consistent in believing that there are some areas of common ground, but also a clear distance between adult and childhood asthma; the experts differ on how much clear distance there is. If asthma is perceived as a single entity which is treated with the same medications as guidelines advocate (12, 13), then there will be an inevitable bias for diagnosis and treatment to be considered mostly homogenous across all ages. Similarly, it is not unexpected that childhood and adult asthma will be considered more heterogenous conditions when perceived from a life course perspective. Perhaps the notable differences are genetics and severe asthma. Hereditary factors are considered to explain up to 70% of asthma causation (14), so from a purely genetic perspective childhood and adult asthma are more different than similar. Severe asthma, as evidenced by persistently poorly controlled asthma despite adequate medication, is vanishingly rare in preschool children and 70% of asthma deaths occurred in individuals whose diagnoses was made in adulthood (15). Recent years have seen the development of targeted therapies such as monoclonal antibodies that are transforming severe asthma management in adults (16).

Perhaps adult and child asthma may be the most useful (or least useless) method of phenotyping asthma. At the time of writing, 3 months after the last article was published, there have been almost 42,000 views of the articles, so the perspectives seem to have struck a chord. Looking forwards, endotypes, and using non-hypothesis driven artificial intelligence may prove more accurate means to tease apart the strands we know are within the heterogenous entity we call asthma. A uniform definition of asthma and a diagnostic test would be very helpful. In the meantime, we carry on diagnosing and managing asthma across the life course, but in the hope that we can do it better in the near future.

## Author Contributions

ST wrote the first draft. ST and JU made meaningful contributions to the final manuscript.

### Conflict of Interest

The authors declare that the research was conducted in the absence of any commercial or financial relationships that could be construed as a potential conflict of interest.
